# The Influence of GLP1 on Body Weight and Glycemic Management in Patients with Diabetes—A Scientometric Investigation and Visualization Study

**DOI:** 10.3390/medicina60111761

**Published:** 2024-10-27

**Authors:** Ileana Pantea, Angela Repanovici, Oana Andreescu

**Affiliations:** 1 Department of Basic, Preventive and Clinical Sciences, Transilvania University, 500036 Brasov, Romania; ileana.pantea@unitbv.ro (I.P.); oana.andreescu@unitbv.ro (O.A.); 2 Faculty of Product Design and Environment, Transilvania University, 500036 Brasov, Romania

**Keywords:** body weight, dulaglutide, semaglutide, diabetes, scientometric analyses

## Abstract

Diabetes medications can affect weight and cardiovascular health. Some medications can aid in weight management, while others may lead to weight gain. Patients must be monitored and receive appropriate care to manage weight and prevent cardiovascular complications. Despite advancements in diabetes treatments that can influence weight and cardiovascular outcomes, ongoing research is necessary in this intricate field. Long-term effects, individual variations, and combination therapies are still subjects of uncertainty and ongoing investigation. The major objective of the research is to evaluate the impact of glucagon-like peptide-1 receptor agonists (GLP-1 RAs) on body weight in diabetic patients through a scientometric assessment. Methodology: Research data were gathered from the Web of Science Core Collection (WoSCC) database by searching for the keywords “Body Weight”, dulaglutide, and semaglutide, identifying 60 relevant articles in the field. While there are advantages in managing diseases in which the cardiovascular system is implicated, there are also clinical considerations for personalized medicine and shared decision-making. The scientometric analysis of the articles revealed important insights into how dulaglutide and semaglutide impact weight management and their potential benefits for managing cardiovascular diseases in individuals with diabetes. Conclusions: Semaglutide shows superior outcomes compared to other commercially available GLP-1RAs, particularly in improving blood sugar control, lowering body weight, and addressing other cardio-metabolic risk factors in individuals with type 2 diabetes (T2DM). The findings suggest that GLP-1 RAs have the potential to provide cardiovascular protection by influencing various physiological factors such as blood pressure, pulse rate, glycated hemoglobin (HbA1c) levels, and the urinary albumin-to-creatinine ratio (RAC). The development and validation of the 4GI model provides a sophisticated tool for evaluating the complex interactions involved in diabetes treatments, offering insights into the mechanisms of action of various medications.

## 1. Introduction

Diabetes is a chronic and progressive metabolic disorder characterized by high levels of glucose in the bloodstream. If not adequately managed, this condition can cause serious complications, including cardiovascular disease, neuropathy, nephropathy, and retinopathy [1].

In addition, diabetes places a significant burden on both individuals and healthcare systems around the world, both in terms of clinical care and economic costs. The management and prevention of diabetes and its complications are of the utmost importance to public health.

There exists a spectrum of pharmacotherapy options accessible for patients who have type 2 diabetes (T2DM). These options can be broadly categorized into oral medications and injectable medications. Regarding oral medication, there are metformin, dipeptidyl peptidase-4 inhibitors (DPP4is), thiazolidinediones, sulfonylureas, and sodium-glucose cotransporter-2 inhibitors (SGLT2is), and regarding injectable medications, there are insulins and glucagon-like peptide-1 receptor agonists (GLP-1 RAs) [1,2].

**Figure 1:**
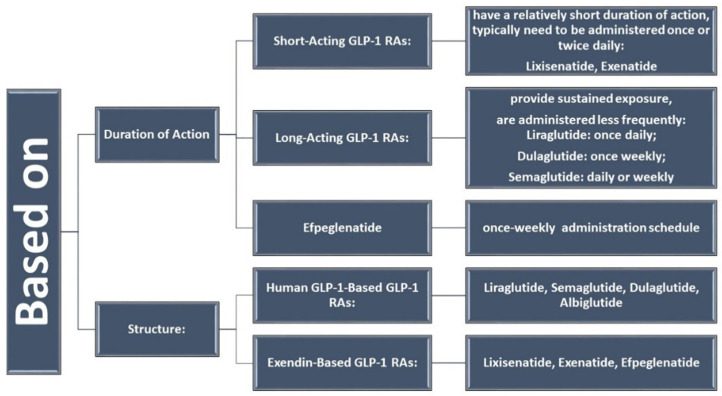
Classification of GLP-1 RAs.

Certainly, GLP-1 RAs are a valuable class of medication used in the management of type 2 diabetes. GLP-1 RAs have an important role when talking about the treatment of type 2 diabetes by addressing both blood sugar control and weight management [3]. GLP-1 RAs have unique qualities such as the duration of action, frequency of dosing, and mechanism of action, allowing healthcare professionals to customize treatment plans according to the individual needs and preferences of the patient [4,5,6]. Not only do these medications have a positive impact on glycemic control, weight management, and cardiovascular outcomes, but they also provide an array of options for optimizing diabetes management based on the specific profiles of patients, as demonstrated by research conducted by Desouza [7]. The classification of GLP-1 RAs is shown in Figure 1.

Each of these GLP-1 RAs may have slightly different properties and characteristics, which can influence their clinical use and patient preferences [8]. Healthcare providers carefully evaluate various factors when determining which is the most appropriate GLP-1 RAs for individual patients with type 2 diabetes [9,10].

## 2. Literature Review Using Scientometric Methods

Scientometric studies represent investigations that analyze and measure scientific production and the impact of articles and published research. This research focuses on the use of quantitative and statistical methods for evaluating and quantifying certain aspects of an activity, providing information about general tendencies in a field and the evolution of research in time [11,12].

The authors opted for the use of the Web of Science database for scientometric research due to the wide-range coverage of scientific publications. Web of Science is recognized as one of the most comprehensive bibliographic databases, including a variety of prestigious journals. The quality and reliability of data are secured through rigorous selection, making the information reliable and relevant for scientometric research. Additionally, the database provides a choice of advanced filtering, sorting, and data visualization, which contributes to the effectiveness of the analysis [9,13].

The strengths of the study using Web of Science are as follows.

Access to Extensive and Relevant Data: Web of Science provides a comprehensive database with a large volume of relevant articles and information.

Visual Analysis: VOS viewer version 1.6.20 allows for the creation of visual maps, highlighting relationships between concepts, authors, and publications, making it easier to identify trends and collaboration networks.

Identification of Trends and Gaps: the study highlights research trends and identifies gaps in the existing literature, offering directions for future research.

The limitations of the study are as follows. 

Database Bias: Web of Science may have a selection bias, including more articles from certain journals or regions, which can limit the generalizability of the results.

Software Limitations: Vos Viewer has its own limitations regarding the complexity of the analysis and interpretation of the results. Visual maps can sometimes be difficult to interpret without adequate context.

Focus on Citations: Scientometric analysis heavily relies on citations, which do not always reflect the true quality or relevance of the research. Highly cited articles are not necessarily the most innovative or useful.

Rapid Evolution of the Field: the medical research field, including diabetes research, evolves rapidly.

Subjective Interpretation: the interpretation of visual maps and data can be subjective and influenced by the analysts’ perspectives and knowledge.

The VOS viewer represents an application free of charge and user-friendly for the visualization and review of scientific networks. This program is useful for generating maps and graphical visualizations of co-citation, term co-citation, or author co-citation networks. The VOS viewer has advanced data analysis functionality, including identifying groups and communities within networks, analyzing key terms and associated terms, and investigating the evolution of networks over time [10,14].

## 3. Research Methodology

The research questions addressed the influence of diabetes medications on weight management and their benefits on associated conditions, particularly cardiovascular disease, to investigate this question. Two drugs from new classes with multiple effects, not only on blood glucose but also on weight and other associated cardiovascular diseases, were selected: dulaglutide and semaglutide.

**Figure 2:**
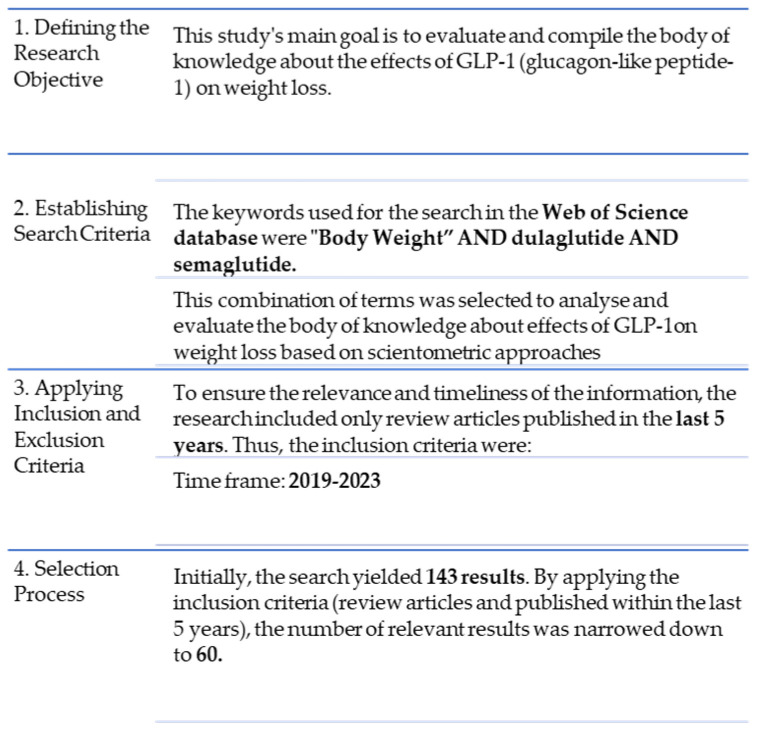
The methodology’s key steps.

A scientometric approach was employed to synthesize the latest advancements in the field, explore current topics, and identify future development trends. The Web of Science Core Collection was utilized to retrieve relevant literature and articles using the search phrase “Body Weight” AND dulaglutide AND semaglutide, resulting in the identification of 60 pertinent articles. The methodology is shown in Figure 2.

## 4. Results

The research team downloaded the database of 60 identified articles and used VOS viewer to analyze the key terms and associated terms within a network. This analysis provided a comprehensive understanding of the themes and relationships present in the literature, shedding light on the influence of dulaglutide and semaglutide on weight balance and associated conditions in diabetes patients. The results included 233 keywords, and the inclusion criteria in the study were that each keyword appeared at least twice. Thus, 90 keywords were identified and distributed in six distinct clusters.

**Figure 3:**
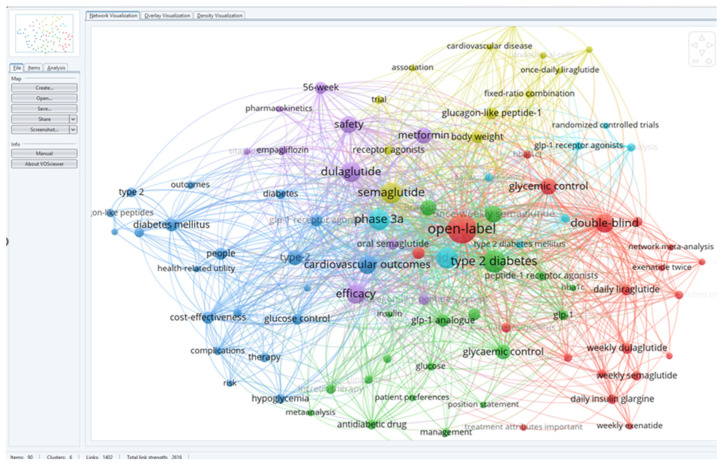
Visualization of clusters.

For a better visualization of the clusters and relationships between the terms, one can refer to Figure 3.

**Figure 4:**
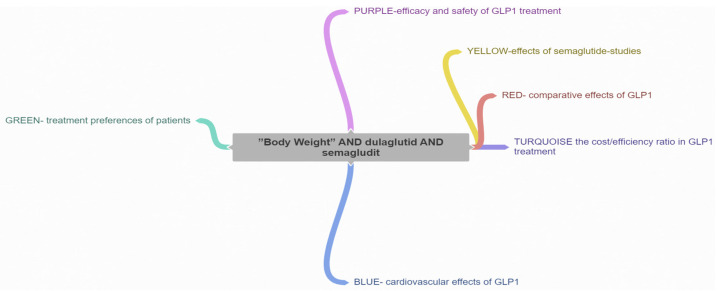
Research directions—results from the clusters analysis.

Following a detailed analysis of each cluster, six distinct research directions were identified in the studied field and the articles corresponding to each cluster (Figure 4).

The research team assigned 60 articles to the six clusters, depending on the topic developed in each cluster. There was a review process of articles associated with each group, aiming at developing and refining the identified research directions. In the review process, it was found that 16 articles did not fit the established research directions, and, subsequently, they were excluded from the analysis. Thus, 44 relevant articles remained in the research for the analysis and synthesis of the information within the identified research directions.

1. BLUE cluster focuses on the cardiovascular effects of GLP-1 RAs [3,4,6,15,16,17]
2. YELLOW cluster explores the effects studied for semaglutide [15,18,19,20,21,22,23,24]
3. RED cluster approaches the comparative effects of GLP-1RAs [19,25,26,27,28,29,30,31,32,33,34]
4. GREEN cluster investigates patient treatment preferences [8,35,36,37,38]
5. PURPLE cluster deals with effectiveness and safe treatment with GLP-1Ras [7,19,25,32,39,40,41]
6. TURQOUISE cluster analyzes the cost/efficiency ratio in GLP-1RAs treatment [19,26,42,43,44,45,46]

## 5. Cluster Analysis

### 5.1. C1: BLUE—Cardiovascular Effects of GLP-1Ras (See Figure 5)

The newfound knowledge that the GLP1-RAs with longer action have the potential to lower the risk of cardiovascular events has sparked curiosity in knowing the mechanisms behind cardiovascular protection. In a meta-analysis of six trials, exenatide administered twice daily was found to decrease systolic blood pressure by 2–4 mmHg when compared with insulin or a placebo [5]. However, this reduction in blood pressure did not correspond to changes in key markers of the renin-angiotensin-aldosterone system (RAAS) or sodium balance. This suggests that the mechanism by which exenatide reduces blood pressure might be independent of the RAAS or alterations in sodium handling. Exenatide offers a modest reduction in systolic blood pressure without affecting RAAS components or sodium excretion. The finding that systemic vascular resistance remains unchanged with exenatide despite the potential vasodilatory effects of GLP-1RAs suggests that the mechanism behind the observed reduction in systolic blood pressure is complex and likely involves factors beyond direct vascular resistance modulation. While it is clear that GLP-1RAs have positive effects on cardiovascular health, particularly by reducing plaque burdens and increasing plaque stability, the precise mechanism remains an area of active research [3]. These drugs are thought to work through a combination of reducing inflammation, improving lipid profiles, enhancing endothelial function, and stabilizing smooth muscle cell proliferation. The multifactorial impact slows down the development and progression of arteriosclerosis, reducing the risk of acute cardiovascular events and improving long-term cardiovascular health. Further studies are needed to fully elucidate these mechanisms and to optimize the use of GLP-1RAs in cardiovascular disease management. GLP-1RAs can improve endothelial function, increase nitric oxide synthesis, and enhance vasodilatation. The full impact on vascular function is not yet fully understood. Variability in study results, differences in patient populations, and the complexity of vascular health contribute to this uncertainty. Further research, particularly long-term studies and those focusing on specific patient populations, is needed to clarify the vascular effects of GLP-1RAs. Liraglutide and semaglutide, two GLP-1RAs, have been shown to significantly reduce postprandial excursions of triglycerides and apolipoprotein B48 (ApoB48) in patients with type 2 diabetes mellitus following a fat-rich meal. This effect is noteworthy because it occurs independently of gastric emptying rates, suggesting that these drugs influence lipid metabolism through mechanisms other than simply slowing digestion. This is likely achieved through the attenuation of intestinal lipoprotein production contributing to better lipid management and potential cardiovascular protection. The anti-inflammatory effects of GLP-1RAs, along with their ability to improve endothelial function and lipid profiles, may indeed stabilize atherogenic plaques, reduce the risk of acute cardiovascular events, and attenuate the progression of arteriosclerosis [6]. The combined effect of reducing postprandial hyperlipidemia, lowering chylomicrons, reducing oxidized LDL particles, and decreasing liver fat contributes to the cardioprotective effects of GLP-1RAs. The most prominent GLP-1 RAs in this class have been shown to lower plasma glucose levels to the same degree as insulin regimens. The decrease in two important factors, glycated hemoglobin (HbA1c) levels and the urinary albumin-to-creatinine ratio (RAC), were found to have a combined impact of up to 65% on the observed cardiovascular benefits. The positive cardiovascular outcomes associated with GLP-1RAs as seen in various clinical trials are likely due to their ability to delay the arteriosclerotic process. By addressing multiple factors simultaneously, such as inflammation lipid metabolism endothelial function and blood pressure, GLP-1RAs provide a comprehensive protective effect on the cardiovascular system. GLP-1RAs can improve endothelial function, increase nitric oxide synthesis, and enhance vasodilatation. The full impact on vascular function is not yet fully understood. Variability in study results, differences in patient populations, and the complexity of vascular health contribute to this uncertainty [15].

**Figure 5:**
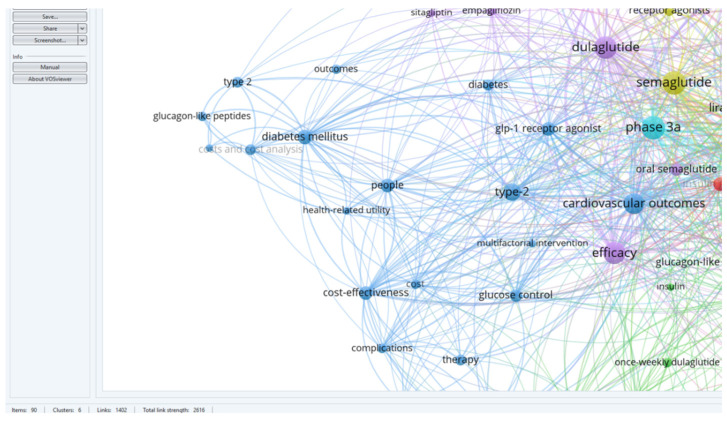
Visualization of cluster one.

### 5.2. C2: YELLOW—Effects of Semaglutide Studies (See Figure 6)

In various clinical trials including both SUSTAIN 7 and AWARD-11 but also SUSTAIN FORTE, the authors used a network meta-regression (ML-NMR) approach. Importantly, the research findings indicated that semaglutide 2.0 mg exhibited higher performance compared to dulaglutide at 3.0 mg and 4.5 mg in the reduction of HbA1c and body weight, as demonstrated by estimated treatment differences (ETDs) and their credible intervals [5]. The investigation did not notice differences in cardiovascular outcomes between the treatments, and it is noted that cardiovascular results for semaglutide at 2.0 mg and dulaglutide at 3.0 mg and 4.5 mg are still pending establishment.

**Figure 6:**
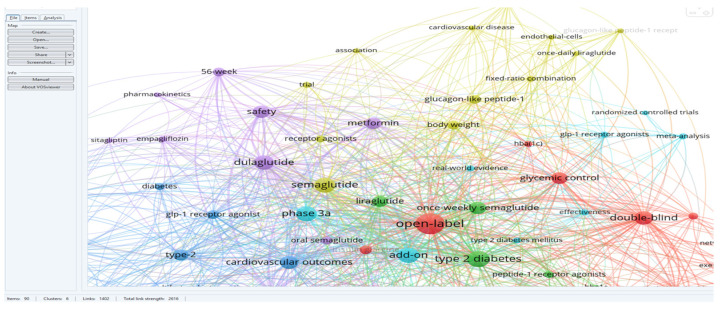
Visualization of cluster two.

Another article explored the effects of switching from semaglutide, a medication used once-weekly, to dulaglutide, another once-weekly medication. The objective of the study was to assess HbA1c levels and body weight during the transition from semaglutide to dulaglutide at various dose levels. The study suggested that switching between these medications, in combination with dose escalation, may offer promising advantages regarding blood sugar management and promoting body weight loss.

Interestingly, semaglutide, which is taken orally, exhibited a better cardiovascular safety profile and a notable decrease in cardiovascular-related death and the overall death rate compared to a placebo medication. Oral semaglutide resulted in a more significant weight loss compared to placebo, sitagliptin, and liraglutide, while demonstrating a similar reduction as empagliflozin [18]. For patients with diabetes and schizophrenia, often coupled with obesity, GLP-1 RAs appear to offer a potential solution to weight reduction. Semaglutide has shown efficiency in reducing HbA1c and body weight in individuals dealing with both type 2 diabetes (T2DM) and schizophrenia.

Although certain GLP-1 RAs, for example exenatide, have shown effectiveness in improving the control of glycemia and reducing body weight in non-schizophrenic populations, the ability of exenatide once a week to reduce body weight in patients with schizophrenia and obesity was found to be insufficient. The inconsistent body weight reduction effects observed with exenatide in a clinical trial imply a possible interaction between exenatide and the dopaminergic signaling system, a system known to influence appetite regulation in the brain reward system [16]. The outcome of the treatment with subcutaneous semaglutide once weekly in lowering HbA1c levels, body weight, and systolic blood pressure has been demonstrated compared to placebo medication and various other antidiabetic medications, offering a more complete understanding of its safety and efficacy profile in real-world settings and the long term [17]. The results of a study conducted by SURE UK in real-world conditions indicated significant improvements in glycemic control, weight reduction, and other crucial clinical factors when using semaglutide as a treatment for a diverse population of patients with type 2 diabetes [17].

Research has been undertaken to investigate the impact of semaglutide taken orally beyond glycemic control and body weight loss. The review will discuss the promising perspective for oral semaglutide in the context of treating not only diabetes but also related conditions like obesity and non-alcoholic fatty liver disease [19]. The renoprotective effects of semaglutide, a GLP-1RA, are currently under investigation in a FLOW study (NCT03819153) [20,21,22]. This study is specifically designed to evaluate how semaglutide affects kidney function in individuals with type 2 diabetes and chronic kidney disease (CKD) [23]. If the FLOW study demonstrates significant renoprotection with semaglutide, it could lead to a shift in the management of patients with type 2 diabetes and CKD, offering a new therapeutic option to slow the progression of kidney disease and reduce the risk of associated complications [24].

### 5.3. C3: RED—Comparative Effects of GLP-1Ras (See Figure 7)

Certain studies focused on the prevalent digestive adverse effects of GLP-1RAs, aiming primarily to assess their tolerability and effectiveness, particularly in terms of once-weekly GLP-1RAs. Additionally, these studies explored the clinical advantages associated with transitioning between various once-per-week GLP-1RAs.

**Figure 7:**
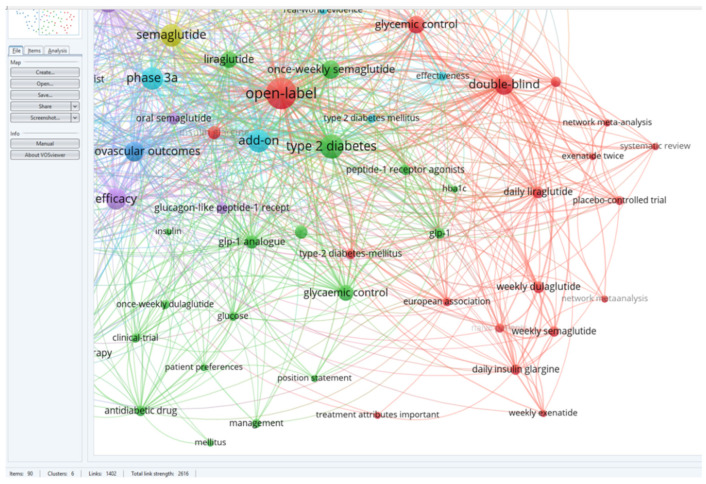
Visualization of cluster three.

Semaglutide had the lowest discontinuation rate, followed by dulaglutide and exenatide [28]. A direct comparison of SUSTAIN 2 and seven clinical trials proved that semaglutide once per week at 0.5 mg but also at 1 mg provides better clinical outcomes compared to sitagliptin at 100 mg and dulaglutide at 1.5 mg [30]. Not all GLP-1 RAs are equal in the treatment of T2DM. Semaglutide seems to offer the most significant efficacy in reducing glucose levels, with liraglutide and dulaglutide following closely behind. If addressing postprandial hyperglycemia is a primary therapeutic objective, agents like lixisenatide or exenatide might be suitable choices due to their effectiveness in this regard. When it comes to weight loss, subcutaneous semaglutide has demonstrated the most substantial results, followed by oral semaglutide and liraglutide. In terms of convenience and ease of use, dulaglutide stands out due to its simple single-use pen delivery system. This convenience is followed by SC semaglutide and liraglutide. The issue of oral semaglutide, administered daily, is yet to be fully determined in comparison to the once-weekly injectable alternatives [25,26].

The analysis of the SUSTAIN trials revealed that a higher percentage of patients achieved both the target of a 1.0% reduction in glycated hemoglobin levels (HbA1c) but also a weight loss of at least 5.0% when treated with subcutaneous semaglutide. This suggests that semaglutide demonstrated superior effectiveness for glycemic control and weight loss compared to the various comparators used in the SUSTAIN trials [33]. A systematic literature review was conducted to identify and compare both the safety and efficacy of semaglutide at a dosage of 14 mg daily with injectable GLP-1RAs [27].

The study conclusion highlighted that the orally administrated semaglutide at a dosage of 14 mg demonstrated significant efficacy, particularly in reducing glycated hemoglobin levels (HbA1c) and facilitating weight loss compared to most of the GLP-1 RAs studied. Some studies focused exclusively on GLP-1RAs that are injectable without including semaglutide [19].

In real-world conditions, it was found that dulaglutide was more effective than liraglutide in reducing levels of HbA1c. The results indicated that dulaglutide influenced reducing HbA1c compared to liraglutide. In real-world situations, the efficacy of dulaglutide and exenatide appeared to be comparable. When selecting among these agents, it is important to consider their variations in both safety and efficacy, while also considering the patient’s individual treatment goals and preferences [28]. There are no notable differences in the risk of hypoglycemia between these agents, while once-a-week exenatide has the lowest risk of causing nausea but also vomiting [35]. Liraglutide has been approved for T2DM and weight management in overweight or obese adults. Orally available GLP-1RAs, including semaglutide and small molecules like TTP273, are also being investigated. In conclusion, GLP-1RAs offer an effective option for managing obesity [29,30,31,32,33].

### 5.4. C4: GREEN—Treatment Preferences of Patients (See Figure 8)

There was no statistically significant distinction in effectiveness between the orally administered semaglutide at 14 mg in comparison to subcutaneous semaglutide at 1.0 mg, although the latter exhibited greater reductions in HbA1c. Affordability is a crucial factor, and assessments have indicated that both subcutaneous and oral semaglutide are more cost-effective, providing smaller costs compared to other GLP-1RAs and oral medications.

**Figure 8:**
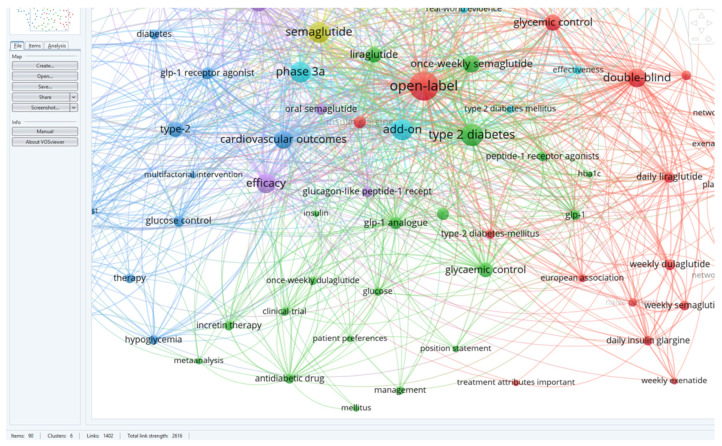
Visualization of cluster four.

The selection between formulations can be customized based on the individual preferences and requirements of the patient [6,34].

Factors such as mode, frequency, and potential effects of administration on body weight are key drivers of patient decision making, and further research on other attributes would be valuable in providing a comprehensive view of patient preferences [34].

### 5.5. C5: PURPLE—Efficacy and Safety of GLP-1RA Treatment (See Figure 9)

Certain characteristics of the patient can affect the administration of GLP-1 RAs. One study aimed to determine whether factors such as age, sex, and diabetes duration but also baseline HbA1c and baseline body mass index (BMI) [35] had an impact on the effects of semaglutide and dulaglutide. Semaglutide is effective in an extended range of patients with T2DM and in its utility in the continuum of care for this heterogeneous population. Oral semaglutide demonstrated noninferiority to injectable semaglutide in lowering HbA1c as well as body weight. Furthermore, it was shown to be superior to placebo medication and “another GLP-1 RA” [42] in terms of “reducing HbA1c and body weight” [11]. GLP-1 RAs do more than just glycemic regulation; they offer cardiovascular protection for the patient, but this may not be uniform for all GLP-1 RAs. Healthcare providers should consider these distinctions when prescribing medications within this drug class. There were only small differences in the clinically significant outcomes of semaglutide effectiveness in populations of various races and ethnic groups [7]. These results help healthcare providers in treatment decisions for diabetic patients regardless of ethnicity [7,8,36,37].

**Figure 9:**
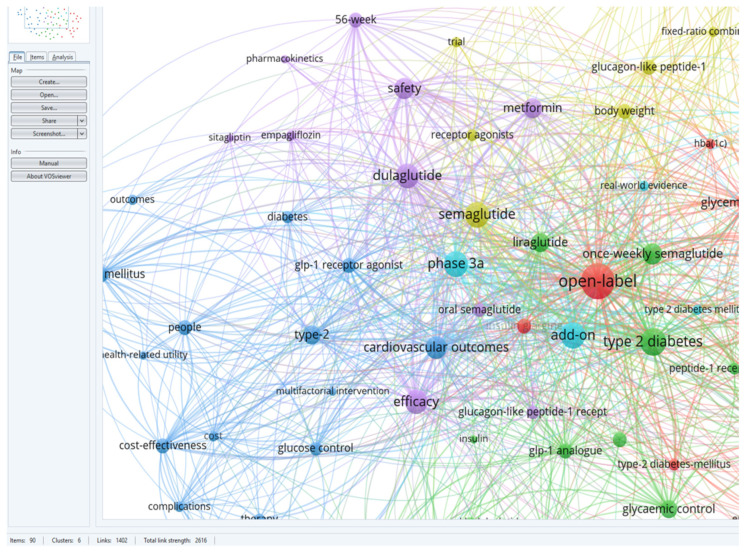
Visualization of cluster five.

A meta-analysis was conducted to systematically evaluate the efficacy of semaglutide once a week by reviewing data extracted from nine randomized clinical trials, indicating that semaglutide is effective and represents a suitable therapeutic method for people with type 2 diabetes, specifically because it is effective in achieving glycemic control and weight management, with minimal cases of hypoglycemia and pancreatitis [36]. Both GLP-1RAs and SGLT2 inhibitors have demonstrated cardiovascular protection in diabetic patients with high risk. The use of GLP-1RAs is not linked with an elevated risk of developing pancreatitis [38].

### 5.6. C6: TURQOUISE—Analysis of the Cost/Efficiency Ratio in GLP-1RA Treatment (See Figure 10)

This effectiveness was observed in the PIONEER phase 3 program, which compared semaglutide with other drugs in a wide range of adults of different ages and with different durations of diabetes, different initial HbA1c levels, different BMI measurements, and additionally, from various racial and ethnic backgrounds. Furthermore, improved tolerability may be observed in individuals with shorter durations of diabetes and younger patients [39].

**Figure 10:**
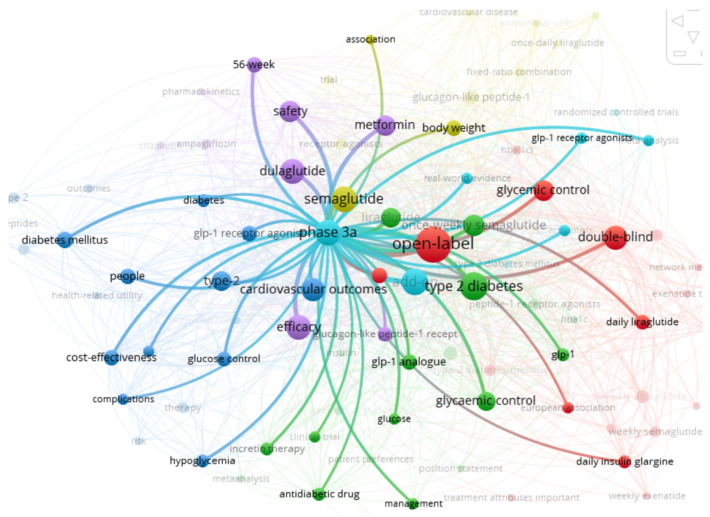
Visualization of cluster six.

The introduction of semaglutide one time per week, either in 0.5 mg or 1 mg doses, provided a better cost-effectiveness for the treatment of individuals in the United Kingdom with type 2 diabetes who did not achieve glycemic control while using metformin. Between 23.4% and 73.3% of patients met the HbA1c target (HbA1c < 7%). Moreover, a range of 25.4% to 69.1% of patients achieved a weight loss of ≥5.0%. Another Danish study aimed to analyze the comparative costs for all forms of injectable GLP-1RAs. The analysis demonstrated that semaglutide provided clinical benefits compared to dulaglutide, extended-release exenatide, liraglutide, and lixisenatide [21].

The development of quantitative pharmacological systems and the appearance of new molecules for the treatment of diabetes could have an important impact on the cost-effectiveness ratio.

Researchers have effectively created and validated an innovative integrated quantitative systems pharmacology (QSP) model, known as the 4GI model. It is designed to quantitatively assess and predict how different drugs, particularly those targeting glucose regulatory mechanisms, influence system dynamics. The 4GI model integrates feedback mechanisms that involve crucial components, for example, glucose, GLP-1 RAs, GIP (glucose-dependent insulinotropic peptide), and insulin. These mechanisms are considered in the context of glucose provocation (such as food intake) and drug interventions [32].

This model serves as a crucial quantitative decision-making tool in drug development. Also, the model is particularly useful in the development of new therapeutic agents targeting the same or related pathways, such as cotaglutide. By providing a quantitative mechanistic understanding of how drugs modulate glucose regulatory pathways, it supports the design development and optimization of new treatment and combination strategies for metabolic disorders. The validated 4GI model is a powerful asset in the advancement of novel therapeutics [24].

The effectiveness and safety of the novel tripeptide GIP molecule were assessed using a Bayesian network meta-analysis in comparison to insulin analogs or selective GLP-1RAs [34]. This analysis was based on clinical trials that evaluated different doses of tripeptide in patients with type 2 diabetes. The analysis indicated that tirzepatide provided favorable efficacy and acceptable safety for patients with type 2 diabetes compared to insulin and GLP-1RAs [32]. In general, semaglutide outperformed dulaglutide at 3 mg with a QALY ratio of £228/QALY gained for total costs compared to dulaglutide at 4.5 mg [40].

The cost effectiveness of semaglutide once a week exceeded that of exenatide or dulaglutide [15]. To achieve clinical objectives, the UK’s National Health Service (NHS) found that semaglutide at 1 mg was significantly more cost-effective than exenatide at 2 mg, dulaglutide at 1.5, and liraglutide at 1.2 mg [28,40]. In terms of effectiveness, semaglutide increased life expectancy by 0.1 years, when compared to empagliflozin at 0.03 years and a 0.03 QALY for dulaglutide [35,41,42,43,44].

Portugal’s healthcare system considers oral semaglutide at 14 mg cheaper for patients with type 2 diabetes with poor glycemic control compared with antidiabetic drugs.

## 6. Discussion

The cardiovascular protection associated with these drugs may be attributed in part to these physiological alterations. Additionally, the decrease in HbA1c levels and urinary albumin-to-creatinine ratios are significant factors contributing to the observed cardiovascular benefits of GLP-1 RAs. It is noteworthy that the cardiovascular benefits of these medications may be linked to their ability to lower plasma glucose levels effectively, comparable to insulin regimens.

In various clinical trials, including SUSTAIN 7, AWARD-11, and SUSTAIN FORTE, a network meta-regression approach was utilized to compare the performance of semaglutide against other GLP-1RAs in reducing HbA1c and body weight.

The trial SUSTAIN 7 compared semaglutide with dulaglutide in patients with inadequately controlled type 2 diabetes. The trial demonstrated that semaglutide at both low and high doses outperformed dulaglutide in terms of glycemic control and weight reduction. This superior efficacy translated into a higher percentage of patients achieving meaningful clinical targets, making semaglutide a powerful tool in the management of type 2 diabetes. Given its similar safety profile to dulaglutide, semaglutide presents a compelling choice for patients and health providers aiming for a more aggressive management of diabetes and associated weight issue. The trial SUSTAIN FORTE compared the efficacy and safety of once-weekly semaglutide at two different doses (2.0 mg vs. 1.0 mg) in adults with inadequately controlled type 2 diabetes who were already on a stable dose of metformin with or without sulfonylurea.

Semaglutide at 2.0 mg offers a valuable treatment option for patients with type 2 diabetes who need a more aggressive management of their condition. It provides superior efficacy in terms of reducing HbA1c and body weight compared to the 1.0 mg dose while maintaining a similar safety profile. This makes the 2.0 mg dose an effective choice for intensifying treatment in patients who require further improvements in glycemic control and weight management. The trial AWARD 11, comparing the efficacy and safety of dulaglutide at doses of 3.0 mg and 4.5 mg vs. 1.5 mg in patients with type 2 diabetes inadequately controlled by metformin, aimed to evaluate whether higher doses of dulaglutide provided additional benefits in terms of glycemic control and weight management while maintaining a comparable safety profile. Escalating the dose of dulaglutide from 1.5 mg to 3.0 mg or 4.5 mg provided clinically relevant dose-related improvements in HbA1c and body weight reduction in patients with type 2 diabetes inadequately controlled by metformin while maintaining a comparable safety profile. Furthermore, oral semaglutide demonstrated a favorable cardiovascular safety profile and significant weight loss compared to other medications, highlighting its potential benefits in managing diabetes and related conditions. Studies focusing on the gastrointestinal adverse effects of GLP-1RAs, particularly once-weekly formulations, have primarily aimed to evaluate the tolerability and effectiveness of these medications. Semaglutide has emerged as a standout option, demonstrating significant reductions in both HbA1c and body weight compared to other medications like exenatide and dulaglutide. Direct comparisons between clinical trials have shown that semaglutide provides better clinical outcomes in terms of glycemic control and weight management. Different GLP-1 RAs exhibit varying efficacy in reducing glucose levels and promoting weight loss, with subcutaneous semaglutide showing the most substantial results. Factors such as convenience and ease of use also play a role in the selection of GLP-1 RAs, with dulaglutide being highlighted for its simple delivery system. The global SUSTAIN clinical trial program has demonstrated the superior effectiveness of semaglutide in achieving glycemic control and weight loss compared to other comparators. The PIONEER phase 3 program compared semaglutide with other medications in a variety of adult groups, demonstrating the efficacy of semaglutide in the treatment of type 2 diabetes mellitus (T2DM). For patients with type 2 diabetes in the UK who are not able to establish glycemic control with metformin, a cost-effective option has been identified: the introduction of semaglutide once weekly in doses of 0.5 mg or 1 mg. Research has indicated that semaglutide medication leads to significant weight loss and different percentages of individuals meeting the HbA1c target. Additionally, the development of the innovative 4GI model, integrating feedback mechanisms related to glucose, GLP-1 RAs, GIP, and insulin, offers a comprehensive understanding of the pharmacological interactions involved in diabetes management.

## 7. Conclusions

Comparing semaglutide with dulaglutide, exenatide, and liraglutide, the findings are as follows.

- Comparison of Efficacy

Our results aligned with the literature (e.g., semaglutide showing greater reductions in HbA1c and weight loss), emphasizing this consistency. Our findings corroborated previous studies, indicating that semaglutide is more effective in achieving glycemic control compared to dulaglutide, exenatide, and liraglutide [16].

- Safety and Tolerability

Consistent with previous research, our results indicated that semaglutide is associated with fewer gastrointestinal adverse events compared to liraglutide, enhancing its appeal as a treatment option. Our findings indicate a higher incidence of side effects, explore possible explanations, and consider the implications for clinical practice [16,45].

- Clinical Implications

Given the superior efficacy and tolerability of semaglutide demonstrated in this study, clinicians may consider it a first-line option for patients with type 2 diabetes seeking optimal glycemic control and weight management [46,47].

Semaglutide seems to show better results compared with the rest of commercially available GLP-1RAs for improving glycemia and other cardio-metabolic risk factors among individuals with T2DM. When considering absolute weight loss, BMI reduction, and the probability of achieving weight losses of >5% and 10%, compared with the baseline, semaglutide is more efficient than dulaglutide, exenatide, and liraglutide, according to current results. The findings suggest that GLP-1 RAs have the potential to provide cardiovascular protection by influencing various physiological factors such as blood pressure, pulse rate, HbA1c levels, and urinary albumin-to-creatinine ratios. While the cardiovascular outcomes of these medications are still under investigation, the promising advantages of switching between semaglutide and dulaglutide suggest potential benefits in managing blood sugar levels but also promoting weight loss. Semaglutide has shown superior efficacy and cost-effectiveness compared to other GLP-1RAs in the treatment of T2DM. The development and validation of the 4GI model provide a sophisticated tool for evaluating the complex interactions involved in diabetes treatment, offering insights into the mechanisms of action of various medications. Through the scientometric analysis of the selected articles, this study uncovered valuable insights into the effects of dulaglutide and semaglutide on weight management and their potential benefits for addressing cardiovascular diseases in individuals with diabetes. The network analysis facilitated a deeper understanding of the interconnected themes and relationships within the research landscape, offering valuable perspectives for future research directions and clinical applications in diabetes management.
